# Evaluation of negative binomial and zero-inflated negative binomial models for the analysis of zero-inflated count data: application to the telemedicine for children with medical complexity trial

**DOI:** 10.1186/s13063-023-07648-8

**Published:** 2023-09-27

**Authors:** Kyung Hyun Lee, Claudia Pedroza, Elenir B. C. Avritscher, Ricardo A. Mosquera, Jon E. Tyson

**Affiliations:** The Institute for Clinical Research and Learning Health Care, The University of Texas Health Science Center at Houston, Houston, TX USA

**Keywords:** Zero-inflated regression model, Count data, Negative binomial, Telemedicine

## Abstract

**Background:**

Two characteristics of commonly used outcomes in medical research are zero inflation and non-negative integers; examples include the number of hospital admissions or emergency department visits, where the majority of patients will have zero counts. Zero-inflated regression models were devised to analyze this type of data. However, the performance of zero-inflated regression models or the properties of data best suited for these analyses have not been thoroughly investigated.

**Methods:**

We conducted a simulation study to evaluate the performance of two generalized linear models, negative binomial and zero-inflated negative binomial, for analyzing zero-inflated count data. Simulation scenarios assumed a randomized controlled trial design and varied the true underlying distribution, sample size, and rate of zero inflation. We compared the models in terms of bias, mean squared error, and coverage. Additionally, we used logistic regression to determine which data properties are most important for predicting the best-fitting model.

**Results:**

We first found that, regardless of the rate of zero inflation, there was little difference between the conventional negative binomial and its zero-inflated counterpart in terms of bias of the marginal treatment group coefficient. Second, even when the outcome was simulated from a zero-inflated distribution, a negative binomial model was favored above its ZI counterpart in terms of the Akaike Information Criterion. Third, the mean and skewness of the non-zero part of the data were stronger predictors of model preference than the percentage of zero counts. These results were not affected by the sample size, which ranged from 60 to 800.

**Conclusions:**

We recommend that the rate of zero inflation and overdispersion in the outcome should not be the sole and main justification for choosing zero-inflated regression models. Investigators should also consider other data characteristics when choosing a model for count data. In addition, if the performance of the NB and ZINB regression models is reasonably comparable even with ZI outcomes, we advocate the use of the NB regression model due to its clear and straightforward interpretation of the results.

**Supplementary Information:**

The online version contains supplementary material available at 10.1186/s13063-023-07648-8.

## Introduction

Zero-inflated (ZI) non-negative count data frequently arise in medical studies, e.g., number of clinic visits, admissions, days in hospital, number of serious illnesses, and medical costs. This data has the following distinct characteristics: (1) the presence of a large proportion of zero values (i.e., zero inflation [1, 2] or sparsity in count data [3, 4]), (2) strictly non-negative values that are right-skewed, and (3) overdispersion (i.e., mean < variance) [5–8]. (Hereafter, we omit the word “non-negative”.) To account for these complexities in this type of data, various models with flexible mixture distributions were introduced over the past decades, including ZI [9, 10] and hurdle regression models [11] using Poisson, quasi-Poisson, negative binomial (NB), and Poisson-Lindley distributions.

While ZI models have been previously compared to their non-inflated counterparts, the conclusions of which model outperforms the other have been inconsistent. For example, Du et al. [12], Connelly et al. [13], and Speedie et al. [14] examined a similar ZI outcome (i.e., number of laboratory tests ordered during a first emergency department visit), but the selected models were not the same. On the basis of the likelihood ratio test, Akaike’s Information Criterion (AIC), and the Bayesian information criterion (BIC), Du et al. suggested that the ZINB model may be favored over Poisson, negative binomial (NB), hurdle, and zero-inflated Poisson (ZIP) regression models. However, the other two studies referred to the NB and hurdle models as “the best fit” and made use of both to predict and explain the outcomes of interest. Choi et al. [15] used a Bayesian model selection criterion to evaluate zero inflation in scRNA-seq datasets. They demonstrated that the primary cause of zero inflation was biological in nature and argued that a quantitative estimate of zero inflation (i.e., an estimate of a parameter accounting for a level of zero inflation in the ZINB distribution) was not a reliable indicator of zero inflation. Outside the medical field, Ver Hoef and Boveng [8] illustrated that the quasi-Poisson produced a better fit for ecological count data when compared to NB based on a diagnostic plot of the empirical fit of the variance. Other studies such as Naya et al. [16] utilized the deviance information criterion and estimates of marginal likelihoods with the method of Newton and Raftery [17] to assess and select the best model among various single- and multi-level ZI regression models. Even though there is a large body of literature on the superiority or non-superiority of ZI models, few studies conducted a further analysis of data characteristics that may directly determine which model(s) give better fit while yielding reliable inferences.

The current study is motivated by a recent clinical trial where, under a Bayesian framework, the ZINB model did not sufficiently outperform the NB model when modeling ZI count outcomes obtained in a trial of children with medical complexity [18]. The ZINB model outperformed both the Poisson and ZIP models; however, the ZIP model did not outperform the Poisson model significantly. The study was a single-center randomized clinical trial, which evaluated the effectiveness of a telemedicine program with comprehensive care (CC) compared to CC alone. For the analysis of this trial, the performance of models (Poisson, NB, and their ZI counterparts) was evaluated using the $k$-fold information criterion (kfoldIC) with $k$equal to 10 (typical value used in studies) [19].

In this simulation study, we re-analyzed the trial data under a Frequentist framework and further investigated which data properties have the largest effect on model fitness under varying sample sizes and degrees of zero inflation. We first compare the model performance of NB and ZINB models based on AIC and then examine whether any characteristics of a ZI outcome influence the model performance and effect sizes. Our hypotheses are as follows. First, we hypothesized that there would be no significant differences between the NB and ZINB regression models in terms of marginal treatment effects, bias, and coverage. Second, we hypothesized that other data characteristics such as skewness and variance of the non-zero part of the data, rather than the number of zero counts in the ZI data and the degree of overdispersion, would be more important in deciding between NB and ZINB models. Last, we hypothesized that the choice of the *best fit* model based on AIC is unrelated to sample sizes.

## Methods

**Fig. 1:**
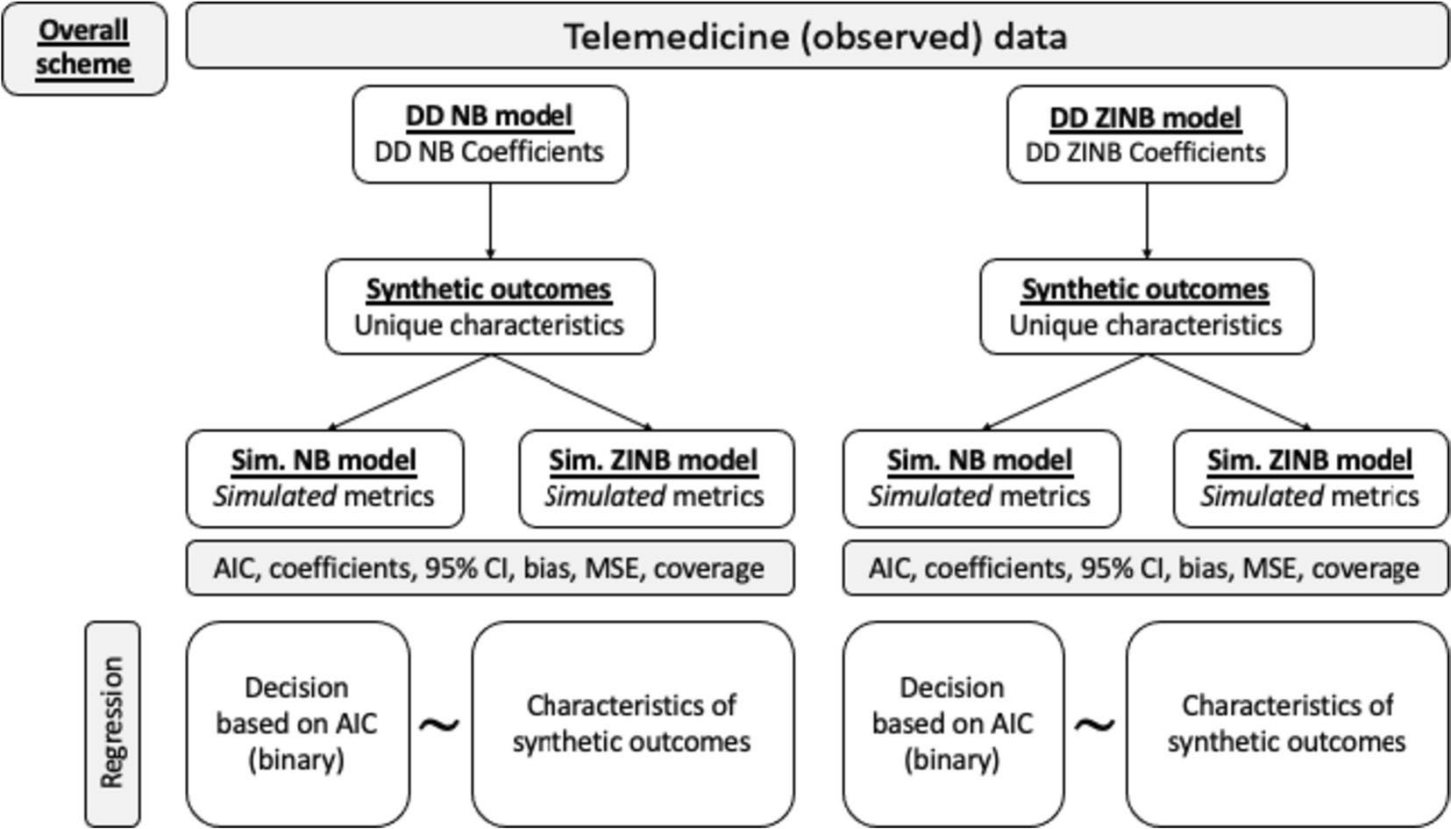
Overall flow chart of the study. We first fitted DD NB and ZINB regression models to the Telemedicine Study dataset. Based on the coefficients from data-derived (DD) models, we generated 5000 synthetic datasets. Unique characteristics of synthetic outcomes were recorded. Synthetic data were fitted with NB and ZINB regression models referred to as sim. models. Each sim. model produced AIC and coefficients of a treatment group variable. Bias and coverage of the treatment coefficient were also calculated. We then created a new dichotomous outcome variable indicating whether the ZINB model gave a better fit to the data based on the AICs from the sim. NB and ZINB models. Using characteristics of the synthetic outcomes as predictors, we performed a ridge logistic regression to identify the important characteristics in determining model preference

The overall scheme of this simulation study is depicted in Fig. 1 (Additional file 1: Figs. S1–S3). Notations used in the study are listed in Table 1.

**Table 1:** Description of notations used in the study (data-derived (DD))

	**Description**	**Types/components**
**DD model**	A regression model based on the observed data. Its coefficients are used to generate the synthetic data	DD NB and ZINB models
**DD coefficient**	Coefficients of a treatment group predictor from a DD model. They serve as a reference value when calculating coverage or bias	Coefficient of treatment group variable (${\beta }_{\mathrm{DD}}$). DD NB and ZINB coefficients
**Synthetic data**	Combination of synthetic outcome and predictors with a size of 422. They are based on the regression coefficients derived from a DD model	Synthetic outcome, synthetic predictors
**Unique characteristics**	Summary statistics of synthetic outcomes. They are used as predictors in a logistic regression model	Overall mean, percentage of zero counts, mean/variance/skewness of the non-zero part, MLEs of $p$ and $r$
**Sim. model**	A regression model fitted to the synthetic data	Simulated NB and ZINB models
**Sim. metrics**	Output of the simulated model	Coefficient of the treatment group, its 95% CI, and AIC
**Modified synthetic data**	Combination of synthetic outcomes and predictors with a total sample size of 60, 80, 100, 200, 600, and 800	Modified synthetic outcome, modified synthetic predictors

### Motivating example

Telemedicine (TM) is an emerging platform for the delivery of health-related services and medical information via telecommunication technology such as computers and smartphones. The COVID-19 pandemic heightened the importance and value of these contactless healthcare services. Previuosly [18], we reported data from a randomized clinical trial comparing CC alone to TM with CC (CC + TM) for medically complex children. There were a total of 422 patients (213 in CC alone and 209 in CC + TM). Using a Bayesian NB regression model with a neutral prior assuming no a priori benefit from TM with CC, we found that the probabilities of a reduction with CC + TM versus CC alone were 99% and 98% for care days outside the home and episodes of serious illnesses, respectively. In this trial, the majority of the outcomes of interest were ZI. Here, we define an outcome as *zero-inflated* if more than 60% of counts are 0 and the outcome is *overdispersed*, which refers to any data in which the variance exceeds the mean. It was interesting to see that, regardless of how much each outcome was ZI, the Bayesian NB model fit most outcomes better than the ZINB model in terms of kfoldIC (with lower numbers representing *better* fit).

In this current study, the primary outcome of interest is the number of serious illness episodes. A serious illness episode was defined as a case in which a patient either had a hospital stay > 7 days was admitted to the pediatric intensive care unit (PICU) or died during the same hospitalization. Approximately 72.7% of the primary outcome values were zero (70% and 75.6% in the CC alone and CC + TM groups, respectively). Here, we also include two secondary outcomes: (1) days in the hospital and (2) care days outside the home. The distribution of days in hospital is similar to the primary outcome, with the exception of a few extreme observations (median 0, interquartile range 0–6, maximum 92). The distribution of care days outside the home differs because the percentage of zero counts is only 5.2%. The characteristics of the three observed outcomes are shown in Table 2. The characteristics include the overall mean, the percentage of zero counts, the mean/variance/skewness of the nonzero part, and the maximum likelihood estimates (MLEs) of two parameters (the shape/stopping parameter, $r$, and the success probability, $p$) for the negative binomial distribution.

**Table 2:** Characteristics of the observed outcomes (Var., variance, % of 0’s, percentage of zero counts) and a list of covariates for adjustment

		**Overall**			**Non-zero part**			**MLE**	
		**Mean**	**Var**	**% of 0’s**	**Mean**	**Var**	**Skewness**	${\varvec{p}}$	${\varvec{r}}$
**Primary**	Serious illness episodes	0.5	1.18	72.75%	1.83	1.90	2.10	0.42	0.36
**Secondary**	Days in hospital	6.73	210.13	52.61%	14.19	338.09	2.36	0.04	0.62
	Care days outside the home	9.0	302.0	5.21%	15.2	306.59	2.79	0.05	0.79

Similar to the original analyses of these outcomes, we include three variables as predictors: (1) treatment group (CC alone = 0; CC + TM = 1); (2) age strata (< 2 years; ≥ 2), (3) baseline risk (risk level 1 [mechanical ventilation], risk level 2 [equal to or above the expected median risk but not ventilator-dependent], and risk level 3 [below the expected median risk]). Length of follow-up (in days) is included as an offset. Using the three outcomes and observed predictors, we performed a NB and ZINB regression analysis. The regression coefficients from the NB and ZINB models were then used as *true* value coefficients to generate *synthetic* data.

### Summary of NB and ZINB distributions

The ZINB distribution is a mixture distribution in which a mass of $p$ (i.e., $0\le p\le 1$) is assigned to excess zeros, while a mass of $\left(1-p\right)$ is assigned to a negative binomial distribution. The NB distribution is also a gamma mixture distribution of Poisson distributions [20], where the Poisson mean $\lambda$ is gamma distributed to account for overdispersion. More specifically, the probability mass function (PMF) of the NB distribution is:

$$\mathrm{Pr}\left(X=x\right)=\frac{\Gamma \left(r+x\right)}{x!\Gamma \left(r\right)}{\left(\frac{r}{r+m}\right)}^{r}{\left(\frac{m}{r+m}\right)}^{x} \mathrm{for}\;x=0, 1, 2, \cdots ,$$

where $m=E(X)$, and $r$, which quantifies the degree of dispersion, is referred to as the *dispersion* or *shape* parameter. In this case, the variance of $X$ is $m+\frac{{m}^{2}}{r}$. Consequently, the PMF of the ZINB distribution is:

$$\mathrm{Pr}\left(Y=y\right)=\left\{\begin{array}{c}p+\left(1-p\right){\left(1+\frac{m}{r}\right)}^{-r} , y=0\\ \left(1-p\right)\frac{\Gamma \left(r+x\right)}{x!\Gamma \left(r\right)}{\left(1+\frac{m}{r}\right)}^{-r}{\left(1+\frac{r}{m}\right)}^{-y} , y=1, 2, 3, \cdots \end{array}\right.$$

The expected count is $E\left(Y\right)=\left(1-p\right)m$, and the variance is $\mathrm{Var}\left(Y\right)=m\left(1-p\right)\left(1+m\left(p+\frac{1}{r}\right)\right)$. Note that the ZINB distribution approaches the ZIP and NB distribution if $r$ tends to $\infty$ and $p$ tends to , respectively.

In the ZINB regression model, the parameters $p$ and $m$ are associated with covariates in the following manner, where $i=1, 2, \cdots ,n$:

$$\mathrm{log}\left({m}_{i}\right)={{\varvec{x}}}_{i}^{\prime}{\varvec{\beta}}\;\;\; \mathrm{and} \;\;\; \mathrm{logit}\left({p}_{i}\right)={{\varvec{z}}}_{i}^{\prime}{\varvec{r}}.$$

Here, ${x}_{i}$ and ${z}_{i}$ are vectors of covariates (or predictor variables) for the NB and logistic components, respectively, where $i$ represents the *i*th of the $n$ independent subjects. In addition, $\beta$ and $r$ are the corresponding vectors of regression coefficients. The regression coefficients are estimated using maximum likelihood estimation. For more details, we refer to Moghimbeigi et al. [21]

### Observed data and data-derived models

NB and ZINB regression models were fitted to the three observed outcomes including the three predictors as covariates in the count model part but only including the treatment group in the logit model part. We refer to these models as the *data-derived* (DD) models. From these models, we obtained estimates of the coefficient for the treatment group variable (${\beta }_{\mathrm{DD}}$; Tables 3 and 4). We refer to them as DD *coefficients* and use them as the true values in our simulation study. To specify the distribution utilized, we append the distribution’s name to the end of DD (e.g., DD NB model, DD ZINB coefficients).

**Table 3:** Regression coefficients from the data-derived (DD) ZINB models used as true parameter values for simulating data

		**DD ZINB model**				
		**NB count model part**				**Logit model part**
		**Treatment group** $({{\varvec{\beta}}}_{\mathbf{D}\mathbf{D}})$	**Age**^**a**^** (** $\ge$** 2 years)**	**Baseline risk**^**b**^** 2**	**Baseline risk**^**b**^** 3**	**Treatment group**
**Primary**	Serious illness episodes	− 0.64	− 0.74	− 0.78	− 1.88	− 15.66
**Secondary**	Days in hospital	− 0.50	− 0.26	− 1.26	− 0.52	− 13.517
	Care days outside the home	− 0.22	− 0.20	− 0.67	− 0.66	0.41

^a^The reference group is age (< 2 years)

^b^The reference group is baseline risk 1

**Table 4:** Regression coefficients from the data-derived (DD) NB models used as true parameter values for simulating data

		**DD NB model**			
		**Treatment group** $({{\varvec{\beta}}}_{\mathbf{D}\mathbf{D}})$	**Age**^**a**^** (** $\ge$** 2 years)**	**Baseline risk**^**b**^** 2**	**Baseline risk**^**b**^** 3**
**Primary**	Serious illness episodes	− 0.49	− 0.74	− 0.74	− 1.86
**Secondary**	Days in hospital	− 0.46	− 0.53	− 0.26	− 1.26
	Care days outside the home	− 0.22	− 0.66	− 0.20	− 0.67

^a^The reference group is age (< 2 years)

^b^The reference group is baseline risk 1

To simulate data, we used both NB and ZINB distributions to evaluate whether the underlying true distribution of the outcome has an effect on the fit of the analysis model. The ZINB regression model comprises two distinct parts: (1) an NB count model part and (2) the logit (binary) model part for predicting excess zeros [22]. A treatment group variable was included in both the binary and NB count model parts, while other covariates for adjustment were employed exclusively in the NB count model part. Thus, the DD ZINB model additionally provided an estimated coefficient for a treatment group variable in the logit part, resulting in the *DD* ZINB coefficients having 2 components.

### Simulation of synthetic data

To obtain the simulated outcomes, two components are required: (1) regression coefficients from the DD models and (2) synthetic predictors (i.e., a treatment group variable and covariates for adjustment). The amount of zero counts in the simulated outcomes reflects both the regression coefficients and the synthetic predictors. Synthetic predictors were generated using three sampling distributions: (1) binomial distribution for the treatment group, (2) multinomial distribution for baseline risk, and (3) truncated normal distribution for age. The values of the parameter(s) in the sampling distributions were generated using a uniform distribution with parameters reflecting the observed data (see Additional file 1: Fig. S2 for details). Based on the synthetic predictors and regression coefficients from the DD models, we generated 5000 sets of the *synthetic* outcomes under both an NB and ZINB distribution. The size of each synthetic dataset was the same as the observed study data, $n=422$. The regression coefficients were obtained using package “glmmTMB” [23] in R (version 4.2.1).

### Sensitivity analysis: sample size

Additionally, we varied the sample size of the synthetic data (60, 80, 100, 200, 600, and 800) to assess whether the results behave differently depending on the sample size. We used the same model parameters and distributions to simulate the *modified synthetic* data. Note that the maximum sample size for this sensitivity analysis was set at 800 because, according to recently published meta-analysis articles [24–28], sample sizes for either pediatrics- or telemedicine-related intent-to-treatment analyses often do not exceed 800. In addition, in this analysis, we excluded cases where the sample size was less than 60. Let us consider a scenario where the sample size is 50 and the percentage of zero counts accounts for 73%, which represents the primary outcome. In such instances, given that the number of non-zero values in the outcome would be 14 or fewer, conducting statistical modeling with only 14 data points can yield results that lack stability. Consequently, in light of this reason, we disregarded the case where the sample size was less than 60.

### Analysis of synthetic data

Each of the three outcomes in the synthetic datasets was analyzed with two different GLMs: NB and ZINB models. For each model, we calculated bias, mean squared error (MSE), and coverage for ${\beta }_{\mathrm{DD}}$. Coverage was estimated based on the 95% confidence intervals (CIs) calculated using the profile likelihood technique, because a normal-based CI (e.g., Wald-type confidence intervals) is known to be imprecise when the sampling distribution of the estimate is non-normal [29]. Note that the identical synthetic datasets were also analyzed with the Poisson and ZIP regression models. However, because their performance in terms of the AIC was significantly worse than that of the NB and ZINB models, we do not report the findings from the Poisson and ZIP regression models here.

### Ridge logistic regression using unique data characteristics as predictors

**Fig. 2:**
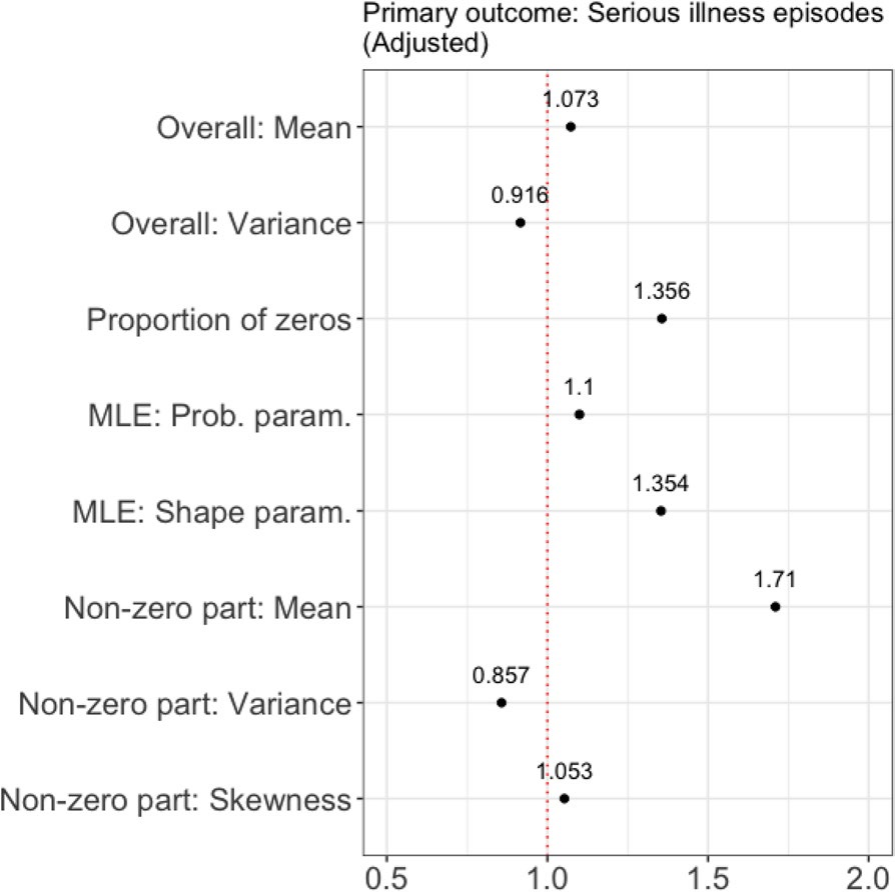
Adjusted odds ratio for a preference for a ZINB model (over an NB model in terms of AIC) regardless of the type of the DD model

We constructed a new dichotomous outcome variable (either 0 or 1) indicating whether the ZINB model gave a better fit to the synthetic dataset based on the AIC from the *simulated* NB and ZINB models. Note that the AIC was chosen over BIC for two reasons. First, the BIC penalizes for sample size [30], which is an unnecessary factor to consider in this case. Second, the BIC evaluates the probability that a model will minimize the loss function (i.e., Kullback–Leibler divergence), whereas the AIC quantifies how good a model is at making predictions. In this regard, the use of the AIC to this study is appropriate. For each synthetic dataset, if the AIC of the ZINB model was lower (i.e., a better fit) than that of the NB model, then 1 was assigned to the new outcome (0, otherwise). Using this outcome variable and 8 unique characteristics of the *synthetic* outcome, we performed a multivariable ridge logistic regression analysis to identify important characteristics significantly associated with the odds of favoring a ZINB model over an NB model (Fig. 2; Additional file 1: Figs. S6 and S7). Specifically, ridge logistic regression was utilized due to the high correlation between predictors (Additional file 1: Fig. S5). Eight unique characteristics of the outcomes were used as predictors: overall mean, overall variance, percentage of zero counts, mean/variance/skewness of the non-zero part, and MLEs of $p$ and $r$ (Table 5; Additional file 1: Table S5). This analysis allowed us to examine how relevant other data aspects are in model selection rather than the amount of zero counts in the ZI data and the degree of overdispersion. For comparison, predictors were standardized to have a mean of zero and a unit standard deviation. For each characteristic, we reported an adjusted odds ratio (OR) obtained from the ridge logistic regression model regardless of which type of DD model was used. We additionally performed the same regression analysis for each DD model as a sensitivity analysis and obtained both adjusted and unadjusted ORs (see Additional file 1: Figs. S6–S14). Furthermore, a simple logistic regression with an unstandardized predictor was performed to present the actual increment or decrement in each predictor corresponding to the OR (see Additional file 1: Table S8).

**Table 5:** Unique characteristics of the synthetic primary outcomes (Var., variance, % of 0’s, percentage of zero counts). Top: simulated from the data-derived (DD) NB model. Bottom: simulated from the DD ZINB model

	**Overall**			**Non-zero part**			**MLE**	
	**Mean**	**Var**	**% of 0’s**	**Mean**	**Var**	**Skewness**	${\varvec{p}}$	${\varvec{r}}$
**DD NB model**								
**Min**	0.15	0.25	48.30	1.27	0.40	1.23	0.08	0.05
**1st Qu**	0.54	1.69	66.10	2.01	3.13	2.33	0.22	0.21
**Median**	0.68	2.52	69.90	2.26	4.78	2.84	0.27	0.26
**Mean**	0.70	2.89	69.90	2.28	5.56	3.07	0.28	0.27
**3rd Qu**	0.84	3.71	73.90	2.52	7.15	3.54	0.34	0.32
**Max**	1.86	15.43	88.20	3.71	31.18	9.39	0.71	0.71
**DD ZINB model**								
**Min**	0.19	0.25	49.10	1.30	0.28	1.21	0.05	0.03
**1st Qu**	0.54	1.66	66.10	2.01	3.15	2.35	0.22	0.21
**Median**	0.68	2.66	69.90	2.26	4.83	2.85	0.27	0.26
**Mean**	0.70	2.96	69.86	2.29	5.74	3.09	0.28	0.27
**3rd Qu**	0.84	3.76	73.30	2.54	7.31	3.56	0.33	0.32
**Max**	1.81	15.71	87.00	3.92	50.77	9.85	0.76	0.63

Note that the Vuong test [31] is commonly applied in practice for demonstrating the appropriateness of a ZI model compared to its non-ZI counterpart. However, the Vuong test is *originally strictly*for comparing two non-nested models [32–34], making it potentially challenging for generalization. For example, NB is nested within ZINB if there is no true zero inflation, and ZIP is nested within ZINB if its dispersion parameter is 0. In addition, it may present a potential bias toward supporting the ZI models, depending on the statistical program used [35]. Despite the fact that some R packages (for example, *nonnest2 *[36]) provide a modified Vuong test that is applicable to both nested and non-nested models, we chose not to use either the original or modified Vuong tests for the abovementioned reasons.

## Results

### Simulation metrics

As shown in Table 5 (and Additional file 1: Table S5), all synthetic outcomes were overdispersed. We first compared *Sim.* NB and ZINB models in terms of *sim.* metrics. Note that we excluded 2.7% and 8.7% of synthetic datasets that caused issues when fitting an NB and ZINB model, respectively. These issues included the inconsistent curvature of the negative log-likelihood surface, an invalid region of parameter space that the optimizer visits, and false convergence. Table 6 summarizes the *sim.* metrics (bias, MSE, and coverage for ${\beta }_{\mathrm{DD}}$) obtained from *sim.* NB and ZINB models for the primary outcome of the number of serious illness episodes. (Additional file 1: Table S2 conveys the same information for the secondary outcomes.) Absolute bias from *sim.* NB and ZINB models are very close regardless of the true underlying distribution. In fact, the *sim*. NB model outperforms *sim.* ZINB model in terms of relative bias and MSE under either true underlying distribution. Coverage is generally at or close to the nominal level (95%), with the exception of the *sim.* NB model under a true ZINB distribution where coverage is 91% (see the “Discussion” section for explanation). The results of the secondary outcome, *days in hospital*, are similar to those of the primary outcome, despite the fact that this secondary outcome was less ZI (53%) and had a considerably higher variance. Note that, regardless of the DD and *sim.* models, bias and MSE were nearly comparable in the case of the other secondary outcome, *care days outside the home*. Regardless of the sample size ranging from 60 to 800, the conclusion remains the same (Additional file 1: Tables S6 and S7).

**Table 6:** Bias, mean squared error (MSE), and coverage for treatment group coefficient, ${\beta }_{DD}$, for the primary outcome (number of serious illness episodes) (DD, data-derived)

	**Primary outcome: number of serious illness episodes**			
	**DD NB model**		**DD ZINB model**	
	**Sim. NB model**	**Sim. ZINB model**	**Sim. NB model**	**Sim. ZINB model**
**Absolute bias**	0.18	0.23	0.20	0.22
**Relative bias**	0.36	0.48	0.31	0.35
**MSE**	0.05	0.09	0.06	0.08
**Coverage**	0.95	0.94	0.91	0.94

### Important predictors of ZINB being preferred

Regardless of the *DD* true model, over 80% of AICs from *sim.* NB models were smaller than those from *sim.* ZINB models. It indicates that the NB model was highly preferred, even with a true ZI outcome (Additional file 1: Table S3). Details of the absolute difference in AICs are shown in Additional file 1: Table S4.

**Fig. 3:**
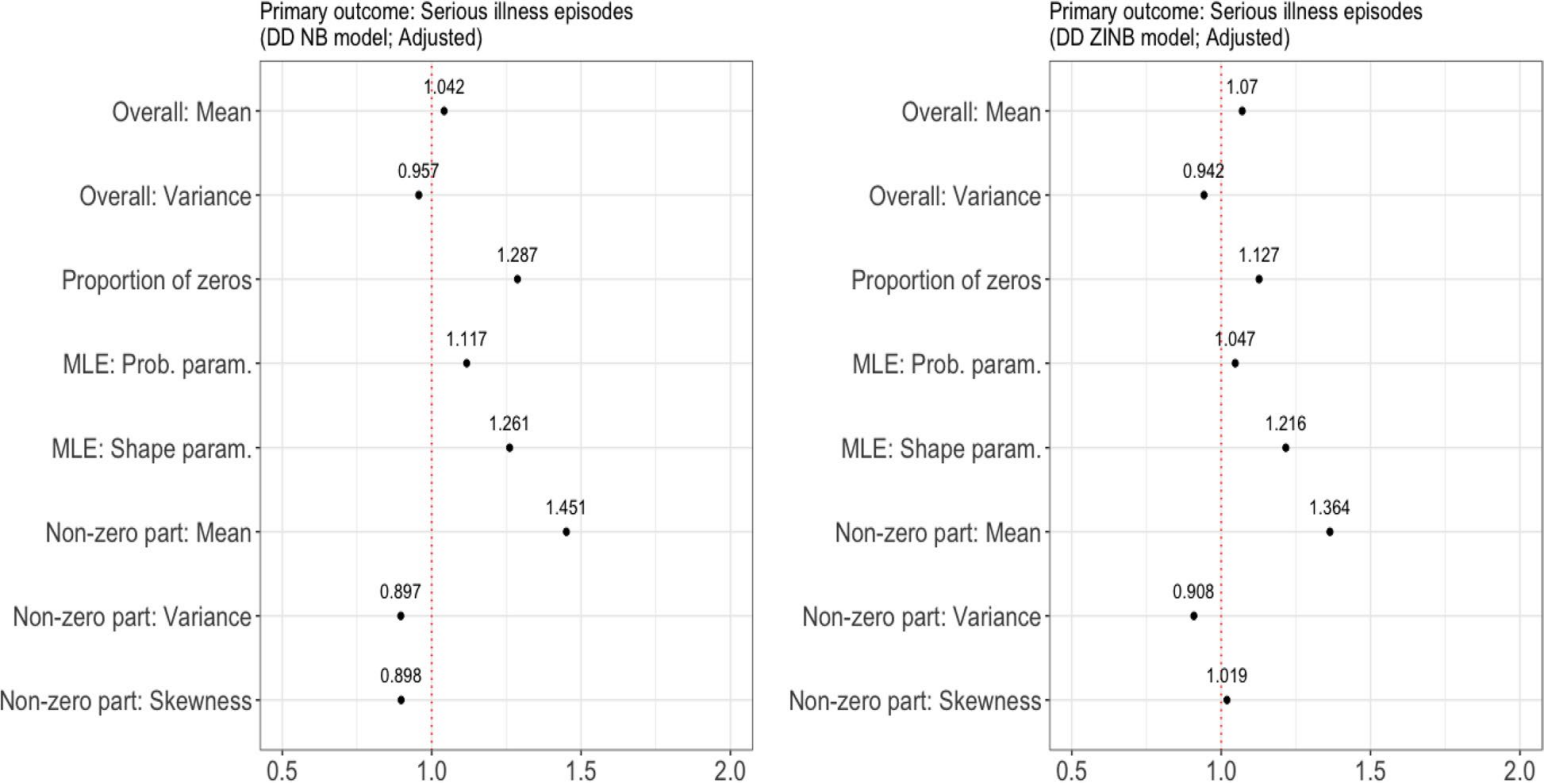
Sensitivity analysis: adjusted odds ratio for a preference for a ZINB model (over an NB model in terms of AIC). Left: based on the results from DD NB models. Right: based on the results from DD ZINB models

The results from the ridge logistic regression analysis indicated that the mean of the non-zero part of the outcome was the strongest positive predictor of a preference for a ZINB model, with OR of 1.71 (Fig. 2). Interestingly, the MLE of the shape, which is defined as a quadratic function of the mean and variance of the outcome, was identified as a predictor as significant as the percentage of zero counts. The same conclusion was reached when the DD NB model was used to generate synthetic primary outcomes (Fig. 3). When the DD ZINB model was used to generate synthetic primary outcomes, the proportion of zeroes played an even smaller role in predicting a preference for the ZINB model than the MLE of the shape parameter. The results of the ridge logistic regression analysis based on the secondary outcomes are provided in Additional file 1: Figs. S9 and S12. With the secondary outcome of days in hospital, where the percentage of zero (52.61%) is less than that of the primary outcome, the odds ratio of the mean of the non-zero part decreased. However, even in this case, the MLE of the shape was a stronger positive predictor of a preference for the ZINB model compared to the percentage of zero counts. Note that the percentage of zero counts was the predictor with the highest odds ratio for the outcome of care days outside of home, where the number of zeros is just 5%. As the percentage of zero increases by 1%, the likelihood of favoring the ZINB model increases by 97%.

## Discussion

The aim of this study was to compare the performance of NB and ZINB regression models in terms of bias, MSE, and coverage and to determine which properties of zero-inflated count data are better described by a ZINB model. Our simulation results indicated that a ZINB regression model does not necessarily outperform an NB model when evaluating ZI medical count outcomes obtained in a trial of children with medical complexity. This is consistent with our original analysis conducted under a Bayesian framework. Even when data were simulated from an underlying ZINB distribution, the NB model had a very similar or even smaller relative bias and MSE for the marginal treatment effect. This suggests that when data is explained and predicted using regression coefficients, as is common in medical and epidemiological studies, there is no significant difference between the NB and ZINB models. Additionally, we want to emphasize that determining the best-fitting model using quantitative model selection criteria (e.g., AIC) is not the only goal of statistical modeling. The ultimate goal of statistical modeling, as Hand [37] stated, is to gain a better understanding of the *real* world. From this perspective, the NB model may be preferred over the ZINB model even when the AIC is worse because the results would be straightforward to interpret.

When comparing which model gave a better fit to simulated data, our results showed that the NB model outperformed that of a ZINB model in terms of bias, MSE, and coverage for the treatment group coefficients, even with outcomes generated from a ZI distribution. It signifies that, in terms of the results (e.g., an intervention effect) in which medical professionals are primarily interested, there is no substantial difference between the ZI and non-ZI regression models, even when the outcome contains excess zeros. Note that, when we used a primary outcome with a sample size of 800, the coverage from the *sim.* NB model under a true ZINB distribution was 0.87, which is lower than that of the *sim.* ZINB model. In fact, for the primary outcome, the coverage decreased as the sample size increased in the combination of the DD ZINB model and the *sim.* NB model (Additional file 1: Table S6), when the sample size ranged between 60 and 800. We note that the corresponding absolute bias and MSE of the NB model decreased (i.e., approaching 0) as the sample size increased. Increasing the sample size typically reduces the width of confidence intervals by lowering the standard error at the same time. As a result, it is possible to achieve a tighter confidence interval, which may result in a low coverage. As shown by Additional file 1: Fig. S4, the interval length of the CIs decreases as the sample size increases. The mean of their lower bounds, in particular, approaches the true coefficient with a smaller standard deviation. This could be the key contributor that brought both bias and MSE close to 0. Further studies investigating the association between data characteristics (e.g., ratio of mean to variance, sample size) and coverage are needed to ensure appropriate model performance.

From the multivariable ridge logistic regression, we observed that the proportion of zeroes played a smaller role in predicting a preference for the ZINB model than the MLE of the shape parameter, when the DD ZINB model was used to generate synthetic primary outcomes. This result indicates that, contrary to popular belief, the percentage of zero counts in predicting a preference for (or fitness of) the ZINB model (over the NB model) is not as substantial as we would assume.

For *care days outside of the home*, bias, MSE, and coverage for the treatment coefficient were comparable regardless of the type of DD and *sim.* models. This is expected given that this outcome was not zero-inflated; hence, there should be no difference between the NB and ZINB models. From the multivariable ridge logistic regression analysis, the percentage of zero counts was the strongest predictor in terms of regression coefficients. In approximately 90% or more cases where the dataset was not suitable for use with the ZI model (i.e., the percentage of zero counts $\approx$ 5%), the NB model exhibited a smaller AIC value, which represents a better fit. However, it is observed that for these particular data, as the percentage of zero counts increases, there is a greater inclination toward favoring the ZINB model. This observation suggests that the prevalence of zero may introduce a bias, leading to a preference for the ZINB model, despite its unsuitability for the given context. This result underscores that when making a choice between the NB model and the ZINB model, it is essential to avoid over-reliance on the percentage of zero counts and instead consider other characteristics of the data. Interestingly, the MLE of the shape parameter in the NB distribution, which is a quadratic function of the mean and variance of the outcome, was the second most important predictor in determining the model preference between NB and ZINB models. This result strongly reinforces our fundamental proposition that when considering the use of ZI regression models, rather than prioritizing the number of zero counts and overdispersion, investigators should consider two-dimensional characteristics such as a shape parameter estimate of an NB distribution, as well as other one-dimensional characteristics of the outcome such as the mean and skewness in the non-zero part of the outcome.

However, there are the following three caveats to consider. First, we exclusively considered the use of the ZINB model (and the ZIP model without any subsequent results described) in this study, which assumes that zero counts are from either the *structural* or *sampling*sources [38]. The scope of this study did not include other ZI models such as the ZI Conway-Maxwell-Poisson model or the ZI generalized Poisson model. The hurdle model, which assumes that all zero counts only originate from the *structural*source, is another popular model for ZI outcomes. This model is similar to the ZI model but may be more versatile as the zero counts can be both deflated and inflated. Depending on the investigators’ subjective opinions and the study objectives, the hurdle model may be a good alternative. It will be worth studying the existence and/or nature of any latent variable(s) that may contribute to the observed ZI count outcome [39]. Second, while various potential scenarios were considered (e.g., sample size, percentage of zero counts, types of DD models), the study was *only* empirically conducted through simulation. A promising extension of this study would be to demonstrate theoretical roles for the mean and skewness of the non-zero part of the ZI outcomes, as well as the MLE of the shape parameter, in an NB or ZINB regression model. Third, we demonstrated these findings by employing medical count outcomes from a single-center trial of children with medical complexity. It should be noted that different types of data may yield different conclusions.

In spite of the caveats discussed, this study is significant because it sheds fresh light on modeling with zero-inflated outcomes, which are frequently observed in medical data. We recommend that the percentage of zero counts in the outcome not be used as the sole and primary reason for selecting ZI regression models. Investigators should also consider other data characteristics such as the mean and skewness of the non-zero part of the outcome when choosing a model for medical count data. In addition, if the performance of the NB and ZINB regression models is reasonably comparable even with ZI outcomes, we advocate the use of the NB regression model due to its clear and straightforward interpretation of the results.

### Supplementary Information

**Additional file 1: Fig. S1.** Detailed flow chart of the observed data analysis (DD: Data-derived). **Fig. S2.** Detailed flow chart of the data simulation (construction of synthetic data). **Fig. S3.** Detailed flow chart of the analysis of simulated data and logistic regression. **Table S1.** Median and interquartile range (IQR) of a treatment group coefficient from the fitted model under two different true models using the observed outcomes (top: number of serious illness episodes; middle: days in hospital; bottom: care days outside the home). **Table S2.** Bias, mean squared error (MSE), and coverage for a treatment group coefficient for the secondary outcomes (top: days in hospital; bottom: care days outside the home). **Table S3.** Percentage of the sim. models that prefer an NB model over a ZINB model. **Table S4.** Details of differences of AIC between sim. NB and ZINB models. **Table S5.** Unique characteristics (median with IQR) of the synthetic secondary outcomes (Var.=variance, % of 0’s = percentage of zero counts). **Table S6.** Sensitivity analysis with different sample sizes (60, 80, 100, 200, 600, 800). The outcome being used is the number of serious illness episodes (primary). Abs. bias: Absolute bias. **Table S7.** Sensitivity analysis with different sample sizes (60, 80, 100, 200, 600, 800). The outcome being used are secondary outcomes (care days outside the home, days in hospital). Abs. bias: Absolute bias. **Fig. S4.** A violin plot of lower (left) and upper (right) bounds of the confidence intervals (CIs) obtained from the sim. NB models under a true ZINB distribution. A blue vertical dotted line represents a DD coefficient (β_DD; -0.64). Mean and standard deviation are represented by the red circle and red solid line in each violin plot. **Fig. S5.** Correlation plot using 8 unique characteristics of data using synthetic primary outcomes (serious illness episodes). Eight unique characteristics of the outcomes include overall mean, overall variance, percentage of zero counts, mean/variance/skewness of the non-zero part, and MLEs of p and r. **Fig. S6.** Adjusted (A) and unadjusted (B) odds ratio for a preference for a ZINB model (over an NB model in terms of AIC) with the primary outcome (serious illness episodes) regardless of the type of DD models. **Fig. S7.** From the DD NB model: Adjusted (left) and unadjusted (right) odds ratio for a preference for a ZINB model (over an NB model in terms of AIC) with the primary outcome (serious illness episodes). **Fig. S8.** From the DD ZINB model: Adjusted (left) and unadjusted (right) odds ratio for a preference for a ZINB model (over an NB model in terms of AIC) with the primary outcome (serious illness episodes). **Fig. S9.** Adjusted (A) and unadjusted (B) odds ratio for a preference for a ZINB model (over an NB model in terms of AIC) with the secondary outcome (care days outside the home) regardless of the type of DD models. **Fig. S10.** From the DD NB model: Adjusted (left) and unadjusted (right) odds ratio for a preference for a ZINB model (over an NB model in terms of AIC) with the secondary outcome (care days outside the home. **Fig. S11.** From the DD ZINB model: Adjusted (left) and unadjusted (right) odds ratio for a preference for a ZINB model (over an NB model in terms of AIC) with the secondary outcome (care days outside the home). **Fig. S12.** Adjusted (A) and unadjusted (B) odds ratio for a preference for a ZINB model (over an NB model in terms of AIC) with the secondary outcome (days in hospital) regardless of the type of DD models. **Fig. S13.** From the DD NB model: Adjusted (left) and unadjusted (right) odds ratio for a preference for a ZINB model (over an NB model in terms of AIC) with the secondary outcome (days in hospital). **Fig. 14.** From the DD NB model: Adjusted (left) and unadjusted (right) odds ratio for a preference for a ZINB model (over an NB model in terms of AIC) with the secondary outcome (days in hospital). **Table S8.** Unadjusted odds ratios with 95% confidence intervals (CIs) based on the unstandardized (raw) predictors regardless of the type of DD models. Asterisk indicates *p*-value < 0.05.

## Data Availability

The trial has been registered at ClinicalTrials.gov (identifier NCT03590509). Deidentified individual participant data will not be made available.

## Abbreviations

ZI: Zero-inflated
NB: Negative binomial
AIC: Akaike’s Information Criterion
BIC: Bayesian information criterion
TM: Telemedicine
DD: Data-derived
CI: Confidence interval
ZINB: Zero-inflated negative binomial
ZIP: Zero-inflated Poisson
CC: Comprehensive care
GLM: Generalized linear model
MLE: Maximum likelihood estimates
MSE: Mean squared error
OR: Odds ratio
