# Supplementary material for: Evaluation of negative binomial and zero-inflated negative binomial models for the analysis of zero-inflated count data: application to the telemedicine for children with medical complexity trial
Source: Trials. 2023 Sep 27;24:613. doi: 10.1186/s13063-023-07648-8 (PMC10523642; doi:10.1186/s13063-023-07648-8)
Supplement: Supplementary file 1 — Additional file 1: Fig. S1. Detailed flow chart of the observed data analysis (DD: Data-derived). Fig. S2. Detailed flow chart of the data simulation (construction of synthetic data). Fig. S3. Detailed flow chart of the analysis of simulated data and logistic regression. Table S1. Median and interquartile range (IQR) of a treatment group coefficient from the fitted model under two different true models using the observed outcomes (top: number of serious illness episodes; middle: days in hospital; bottom: care days outside the home). Table S2. Bias, mean squared error (MSE), and coverage for a treatment group coefficient for the secondary outcomes (top: days in hospital; bottom: care days outside the home). Table S3. Percentage of the sim. models that prefer an NB model over a ZINB model. Table S4. Details of differences of AIC between sim. NB and ZINB models. Table S5. Unique characteristics (median with IQR) of the synthetic secondary outcomes (Var.=variance, % of 0’s = percentage of zero counts). Table S6. Sensitivity analysis with different sample sizes (60, 80, 100, 200, 600, 800). The outcome being used is the number of serious illness episodes (primary). Abs. bias: Absolute bias. Table S7. Sensitivity analysis with different sample sizes (60, 80, 100, 200, 600, 800). The outcome being used are secondary outcomes (care days outside the home, days in hospital). Abs. bias: Absolute bias. Fig. S4. A violin plot of lower (left) and upper (right) bounds of the confidence intervals (CIs) obtained from the sim. NB models under a true ZINB distribution. A blue vertical dotted line represents a DD coefficient (β_DD; -0.64). Mean and standard deviation are represented by the red circle and red solid line in each violin plot. Fig. S5. Correlation plot using 8 unique characteristics of data using synthetic primary outcomes (serious illness episodes). Eight unique characteristics of the outcomes include overall mean, overall variance, percentage of zero counts, mean/varianc [file 13063_2023_7648_MOESM1_ESM.docx]

**Evaluation of negative binomial and zero-inflated negative binomial models for the analysis of zero-inflated count data: Application to the Telemedicine for Children with Medical Complexity Trial**

*Kyung Hyun Lee*^[[1]](#footnote-1)^§*, Claudia Pedroza, Elenir B C. Avritscher, Ricardo A. Mosquera, Jon E. Tyson*

*Center for Clinical Research and Evidence-Based Medicine, Department of Pediatrics, McGovern Medical School, The University of Texas Health Science Center at Houston, Houston, TX*

*Supplementary figures*

Supplementary figure 1 Detailed flow chart of the observed data analysis (DD: Data-derived)


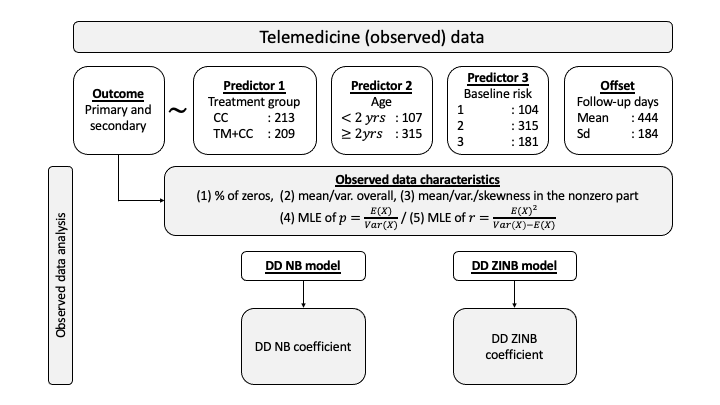


Supplementary figure 2 Detailed flow chart of the data simulation (construction of synthetic data)


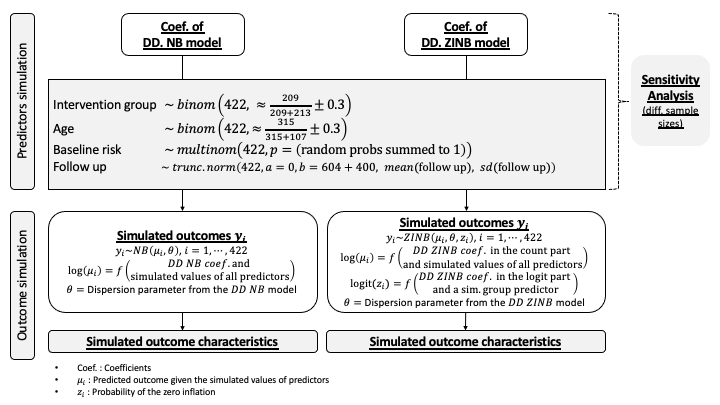


Supplementary figure 3 Detailed flow chart of the analysis of simulated data and logistic regression


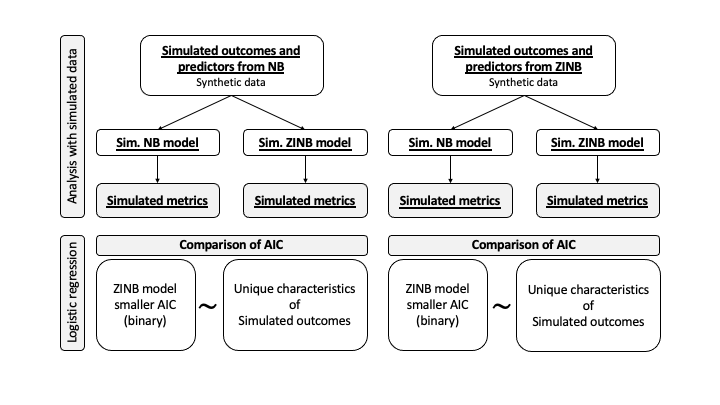


Supplementary table 1 Median and interquartile range (IQR) of a treatment group coefficient from the fitted model under two different true models using the observed outcomes (top: number of serious illness episodes; middle: days in hospital; bottom: care days outside the home)

| **Primary outcome: Number of serious illness episodes** | | | |
| --- | --- | --- | --- |
| **DD NB model** | | **DD ZINB model** | |
| **Sim. NB model** | **Sim. ZINB model** | **Sim. NB model** | **Sim. ZINB model** |
| **Slope (IQR)** | **Slope (IQR)** | **Slope (IQR)** | **Slope (IQR)** |
| -0.49 (-0.63, -0.34) | -0.48 (-0.67, -0.28) | -0.50 (-0.65, -0.36) | -0.62 (-0.80, -0.43) |
|  |  |  |  |
| **Secondary outcome: Days in hospital** | | | |
| **DD NB model** | | **DD ZINB model** | |
| **Sim. NB model** | **Sim. ZINB model** | **Sim. NB model** | **Sim. ZINB model** |
| **Slope (IQR)** | **Slope (IQR)** | **Slope (IQR)** | **Slope (IQR)** |
| -0.46 (-0.63, -0.29) | -0.45 (-0.64, -0.27) | -0.46 (-0.63, -0.29) | -0.49 (-0.67, -0.31) |
|  | | | |
| **Secondary outcome: Care days outside the home** | | | |
| **DD NB model** | | **DD ZINB model** | |
| **Sim. NB model** | **Sim. ZINB model** | **Sim. NB model** | **Sim. ZINB model** |
| **Slope (IQR)** | **Slope (IQR)** | **Slope (IQR)** | **Slope (IQR)** |
| -0.23 (-0.30, -0.15) | -0.23 (-0.30, -0.15) | -0.22 (-0.29, -0.15) | -0.22 (-0.29, -0.15) |

Supplementary table 2 Bias, mean squared error (MSE), and coverage for a treatment group coefficient for the secondary outcomes (top: days in hospital; bottom: care days outside the home)

|  | **Secondary outcome: Days in hospital** | | | |
| --- | --- | --- | --- | --- |
|  | **DD NB model** | | **DD ZINB model** | |
|  | **Sim. NB model** | **Sim. ZINB model** | **Sim. NB model** | **Sim. ZINB model** |
| **Absolute bias** | 0.09 | 0.09 | 0.21 | 0.22 |
| **Relative bias** | 0.38 | 0.39 | 0.41 | 0.44 |
| **MSE** | 0.01 | 0.01 | 0.07 | 0.08 |
| **Coverage** | 0.94 | 0.94 | 0.95 | 0.94 |
|  |  | | | |
|  | **Secondary outcome: Care days outside the home** | | | |
|  | **DD NB model** | | **DD ZINB model** | |
|  | **Sim. NB model** | **Sim. ZINB model** | **Sim. NB model** | **Sim. ZINB model** |
| **Absolute bias** | 0.09 | 0.09 | 0.09 | 0.09 |
| **Relative bias** | 0.38 | 0.38 | 0.39 | 0.39 |
| **MSE** | 0.01 | 0.01 | 0.01 | 0.01 |
| **Coverage** | 0.95 | 0.95 | 0.94 | 0.94 |

Supplementary table 3 Percentage of the sim. models that prefer an NB model over a ZINB model.

|  |  | **In terms of AIC between the *sim.* NB and ZINB models** | | | |
| --- | --- | --- | --- | --- | --- |
|  |  | ***DD* NB model** | | ***DD* ZINB model** | |
|  |  | **Prefer NB** | **Prefer ZINB** | **Prefer NB** | **Prefer ZINB** |
| **Primary** | Serious illness episodes | 88.0% | 12.0% | 80.0% | 20.0% |
| **Secondary** | Days in hospital | 88.3% | 11.7% | 87.1% | 12.9% |
|  | Care days outside the home | 93.3% | 6.7% | 93.3% | 6.7% |

Supplementary table 4 Details of differences of AIC between sim. NB and ZINB models.

|  |  | **AIC of NB – AIC of ZINB** | |
| --- | --- | --- | --- |
|  |  | **DD NB model** | **DD ZINB model** |
| **Primary** | Serious illness episodes | -3.03 (-3.76, -1.61) | -2.48 (-3.52, -0.64) |
| **Secondary** | Days in hospital | -3.06 (-3.76, -1.64) | -2.92 (-3.72, -1.45) |
|  | Care days outside the home | -3.59 (-3.99, -2.51) | -3.58 (-4.00, -2.50) |

Supplementary table 5 Unique characteristics (median with IQR) of the synthetic secondary outcomes (Var.=variance, % of 0’s = percentage of zero counts).

|  |  | **Secondary outcomes** | | | |
| --- | --- | --- | --- | --- | --- |
|  |  | **Days in hospital** | | **Care days outside the home** | |
| **Variable** | | **DD NB model** | **DD ZINB model** | **DD NB model** | **DD ZINB model** |
| **Overall** | Mean | 10.12  (8.44, 11.95) | 10.03  (8.37, 11.90) | 19.10  (16.85, 21.73) | 19.23  (16.87, 21.73) |
|  | Variance | 773.7  (526.1, 1127.2) | 771.4  (516.4, 1141.0) | 584.1  (440.4, 757.4) | 591.4  (441.0, 767.2) |
|  | % of 0’s | 49.10  (46.70, 51.40) | 49.10  (46.90, 49.12) | 5.90  (5.0, 7.1) | 5.90  (5.0, 7.1) |
| **Non-zero part** | Mean | 19.86  (17.13, 22.86) | 19.69  (16.93, 22.74) | 20.34  (18.00, 22.95) | 20.45  (18.11, 23.04) |
|  | Variance | 1326.3  (906.1, 1926.5) | 1313.4  (885.5, 1941.6) | 596.3  (449.9, 774.0) | 603.3  (449.8, 781.8) |
|  | Skewness | 3.94  (3.29, 4.90) | 3.99  (3.32, 4.95) | 2.81  (2.43, 3.31) | 2.81  (2.42, 3.33) |
| **MLE** | $p$ | 0.01  (0.01, 0.02) | 0.01  (0.01, 0.02) | 0.03  (0.03, 0.04) | 0.03  (0.03, 0.04) |
|  | $r$ | 0.14  (0.11, 0.16) | 0.14  (0.11, 0.16) | 0.66  (0.60, 0.73) | 0.66  (0.60, 0.73) |

Supplementary table 6 Sensitivity analysis with different sample sizes (60, 80, 100, 200, 600, 800). The outcome being used is the number of serious illness episodes (primary). Abs. bias: Absolute bias

|  | **DD NB model** | | **DD ZINB model** | |
| --- | --- | --- | --- | --- |
|  | **Sim. NB model** | **Sim. ZINB model** | **Sim. NB model** | **Sim. ZINB model** |
|  | **Abs. bias, MSE, Coverage** | | **Abs. bias, MSE, Coverage** | |
| **60** | 0.52, 0.55, 0.94 | 0.66, 0.87, 0.90 | 0.53, 0.55, 0.92 | 0.65, 0.92, 0.89 |
| **80** | 0.45, 0.38, 0.94 | 0.58, 0.66, 0.88 | 0.46, 0.40, 0.92 | 0.56, 0.73, 0.90 |
| **100** | 0.39, 0.27, 0.93 | 0.51, 0.43, 0.91 | 0.40, 0.27, 0.93 | 0.48, 0.38, 0.92 |
| **200** | 0.27, 0.12, 0.94 | 0.35, 0.20, 0.92 | 0.28, 0.13, 0.92 | 0.33, 0.18, 0.94 |
| **600** | 0.15, 0.04, 0.95 | 0.20, 0.06, 0.94 | 0.19, 0.05, 0.88 | 0.19, 0.06, 0.94 |
| **800** | 0.13, 0.03, 0.95 | 0.17, 0.05, 0.94 | 0.17, 0.04, 0.87 | 0.17, 0.04, 0.95 |

Supplementary table 7 Sensitivity analysis with different sample sizes (60, 80, 100, 200, 600, 800). The outcome being used are secondary outcomes (care days outside the home, days in hospital). Abs. bias: Absolute bias

|  | **Care days outside the home** | | **Days in hospital** | |
| --- | --- | --- | --- | --- |
|  | **DD ZINB model** | | | |
|  | **Sim. NB model** | **Sim. ZINB model** | **Sim. NB model** | **Sim. ZINB model** |
|  | **Abs. bias, MSE, Coverage** | | **Abs. bias, MSE, Coverage** | |
| **60** | 0.24, 0.10, 0.94 | 0.25, 0.10, 0.93 | 0.65, 0.72, 0.93 | 0.70, 0.82, 0.90 |
| **80** | 0.21, 0.07, 0.94 | 0.21, 0.07, 0.93 | 0.53, 0.48, 0.94 | 0.57, 0.54, 0.91 |
| **100** | 0.18, 0.05, 0.94 | 0.18, 0.06, 0.93 | 0.47, 0.36, 0.94 | 0.49, 0.40, 0.91 |
| **200** | 0.12, 0.02, 0.95 | 0.13, 0.03, 0.95 | 0.31, 0.16, 0.94 | 0.33, 0.17, 0.93 |
| **600** | 0.07, 0.01, 0.95 | 0.07, 0.01, 0.95 | 0.17, 0.05, 0.95 | 0.18, 0.05, 0.94 |
| **800** | 0.06, 0.01, 0.95 | 0.06, 0.01, 0.95 | 0.15, 0.04, 0.94 | 0.16, 0.04, 0.94 |

*Supplementary figure 4 A violin plot of lower (left) and upper (right) bounds of the confidence intervals (CIs) obtained from the sim. NB models under a true ZINB distribution. A blue vertical dotted line represents a DD coefficient (*$\beta_{DD}$*; -0.64). Mean and standard deviation are represented by the red circle and red solid line in each violin plot.*


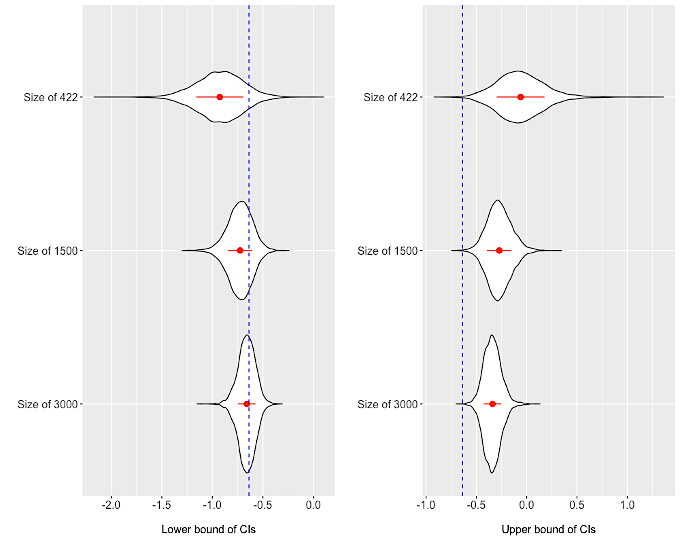


Supplementary Figure 5 Correlation plot using 8 unique characteristics of data using synthetic primary outcomes (serious illness episodes). Eight unique characteristics of the outcomes include overall mean, overall variance, percentage of zero counts, mean/variance/skewness of the non-zero part,
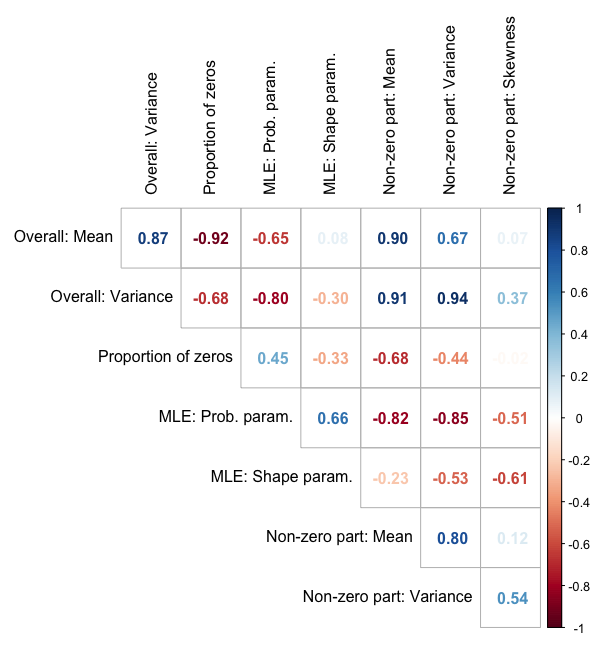
and MLEs of p and r.

Supplementary Figure 6 Adjusted (A) and unadjusted (B) odds ratio for a preference for a ZINB model (over an NB model in terms of AIC) with the primary outcome (serious illness episodes) regardless of the type of DD models.


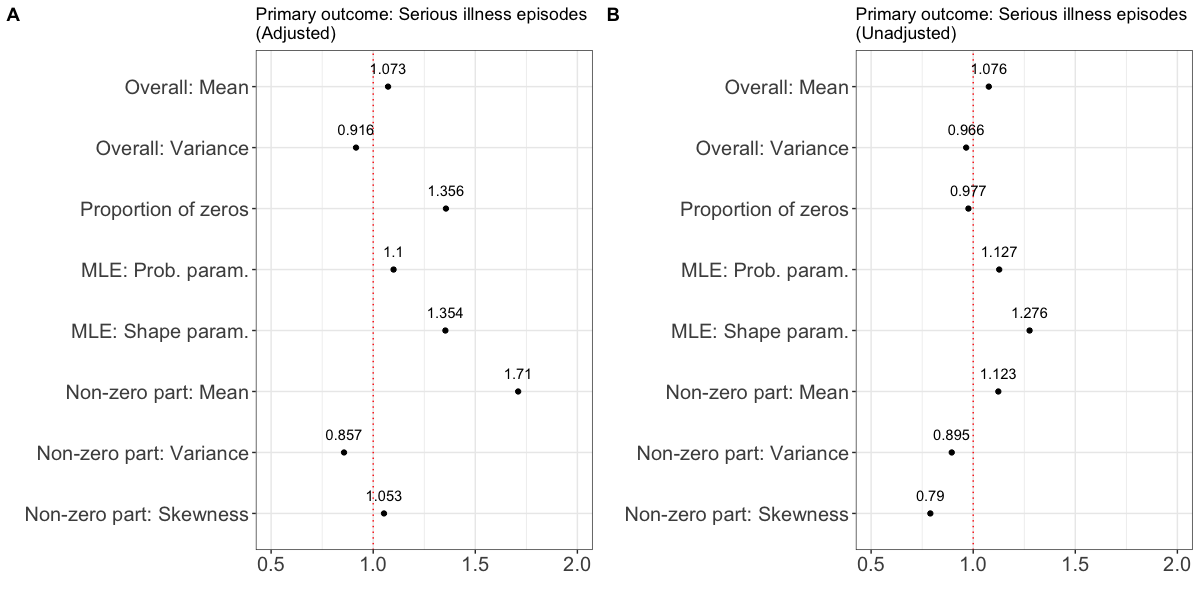
Supplementary Figure 7 From the DD NB model: Adjusted (left) and unadjusted (right) odds ratio for a preference for a ZINB model (over an NB model in terms of AIC) with the primary outcome (serious illness episodes)

Supplementary Figure 8 From the DD ZINB model: Adjusted (left) and unadjusted (right) odds ratio for a preference for a ZINB model (over an NB model in terms of AIC) with the primary outcome (serious illness episodes)


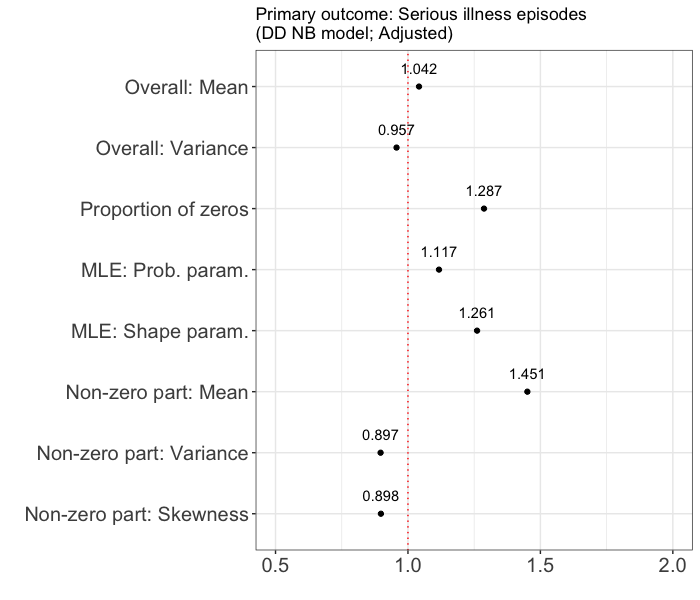

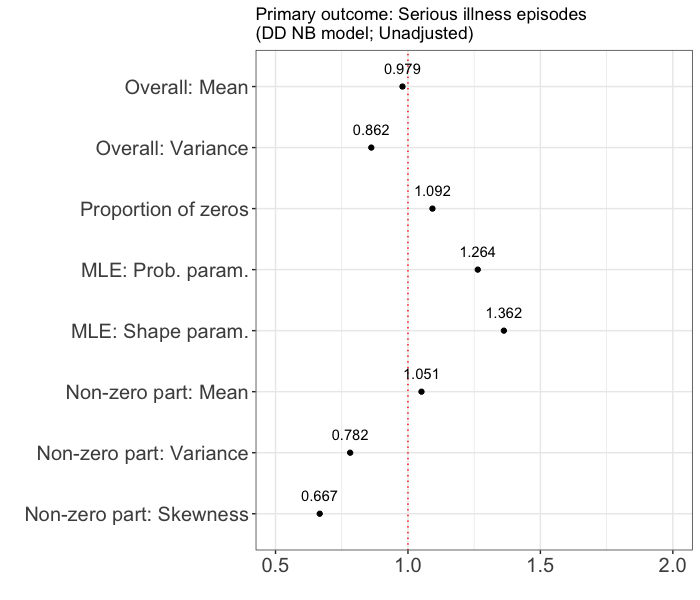


Supplementary figure 9 Adjusted (A) and unadjusted (B) odds ratio for a preference for a ZINB model (over an NB model in terms of AIC) with the secondary outcome (care days outside the home) regardless of the type of DD models.


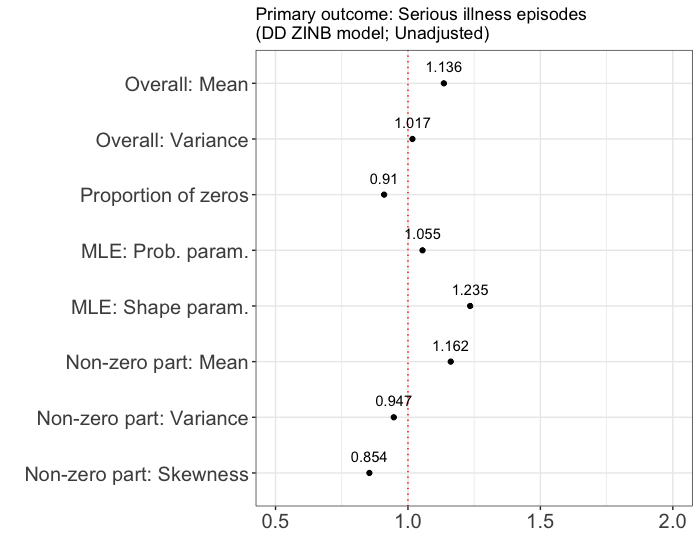

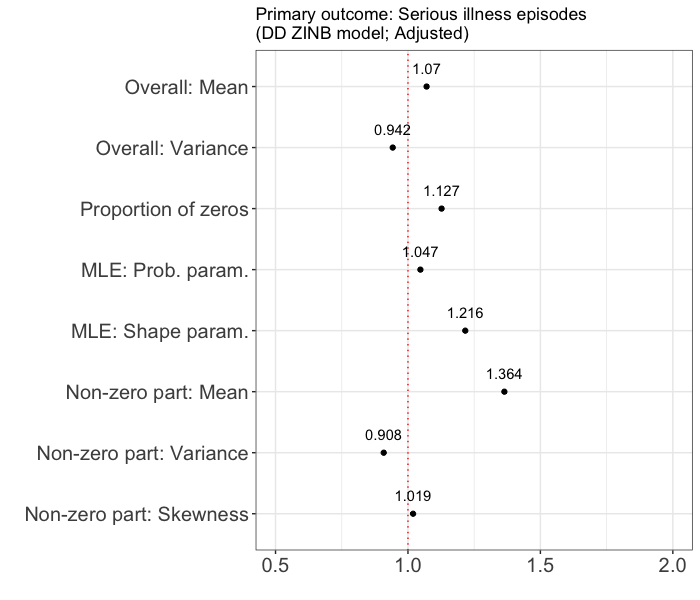


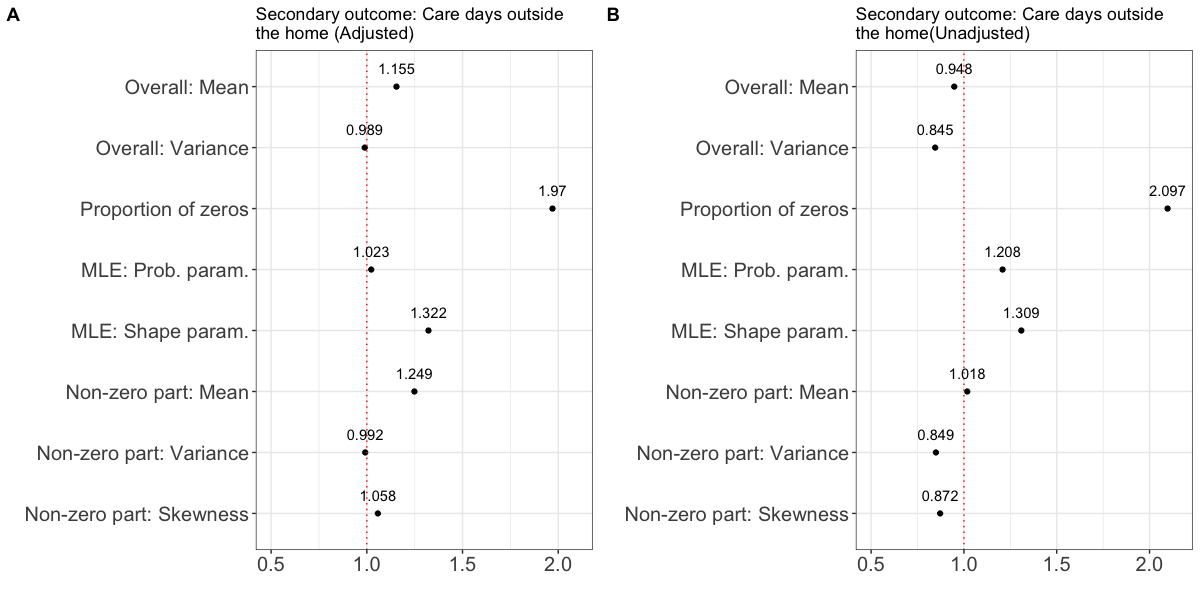


Supplementary Figure 10 From the DD NB model: Adjusted (left) and unadjusted (right) odds ratio for a preference for a ZINB model (over an NB model in terms of AIC) with the secondary outcome (care days outside the home


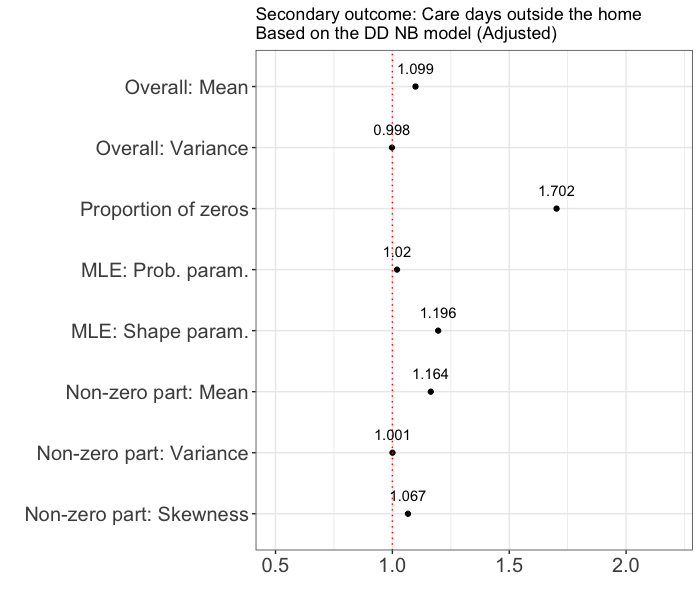

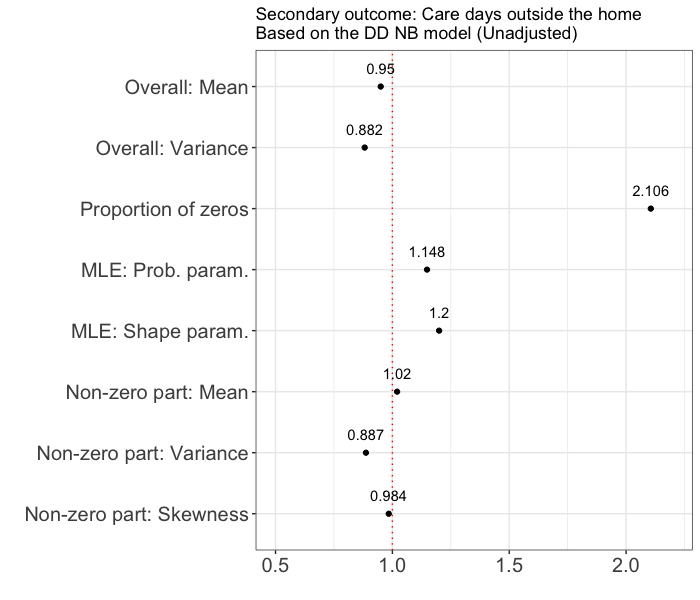


Supplementary figure 11 From the DD ZINB model: Adjusted (left) and unadjusted (right) odds ratio for a preference for a ZINB model (over an NB model in terms of AIC) with the secondary outcome (care days outside the home


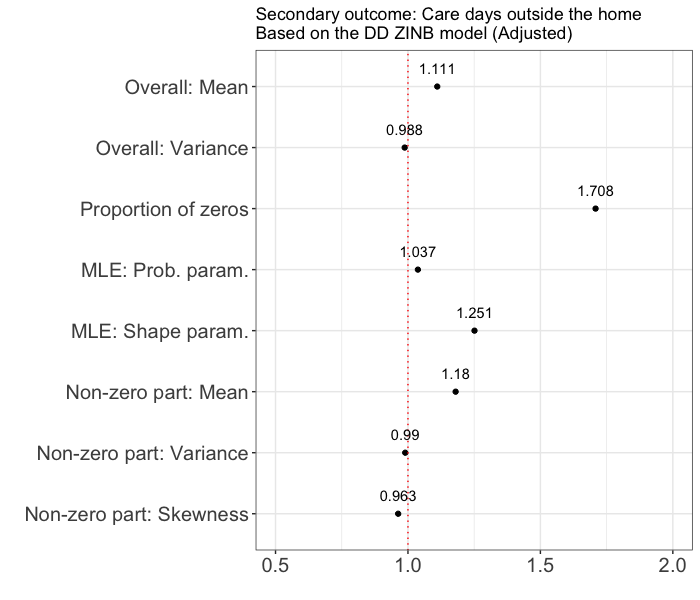

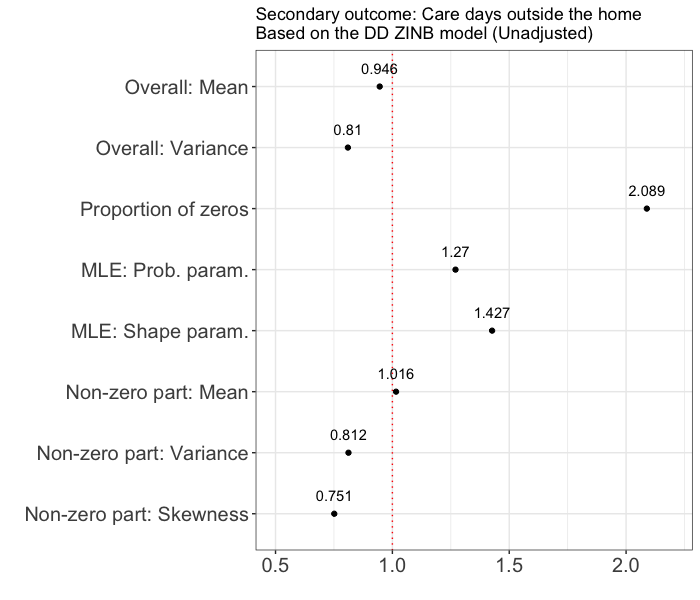


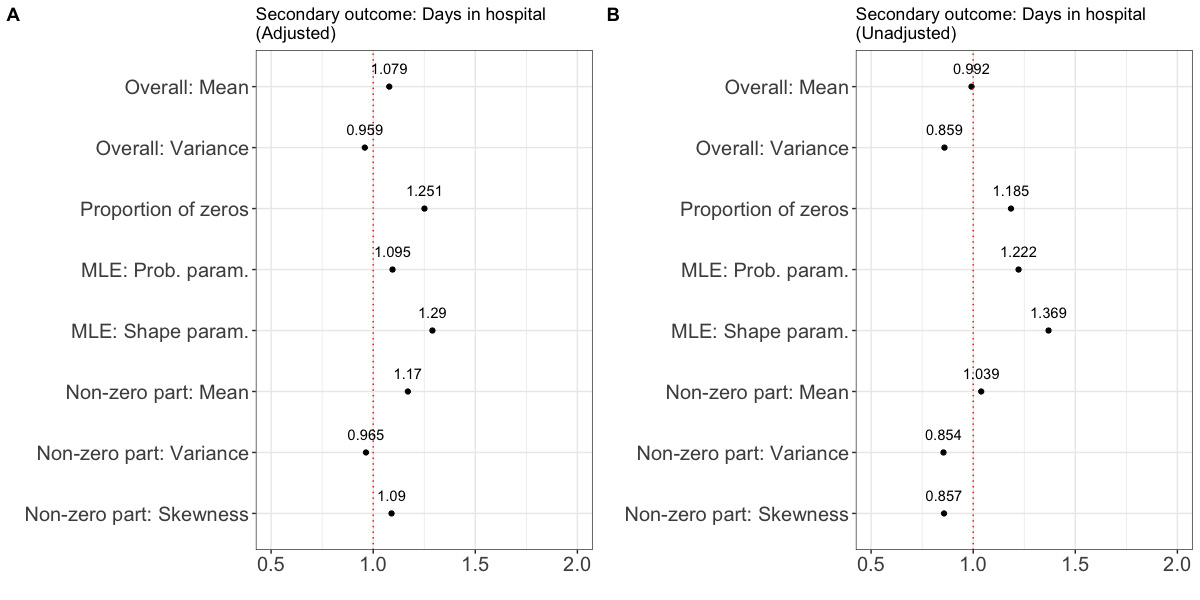
Supplementary Figure 12 Adjusted (A) and unadjusted (B) odds ratio for a preference for a ZINB model (over an NB model in terms of AIC) with the secondary outcome (days in hospital) regardless of the type of DD models.

Supplementary Figure 13 From the DD NB model: Adjusted (left) and unadjusted (right) odds ratio for a preference for a ZINB model (over an NB model in terms of AIC) with the secondary outcome (days in hospital)


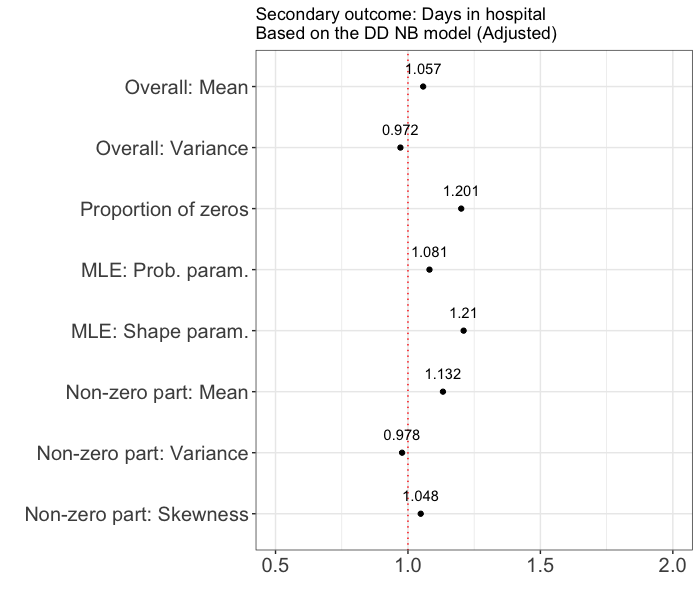

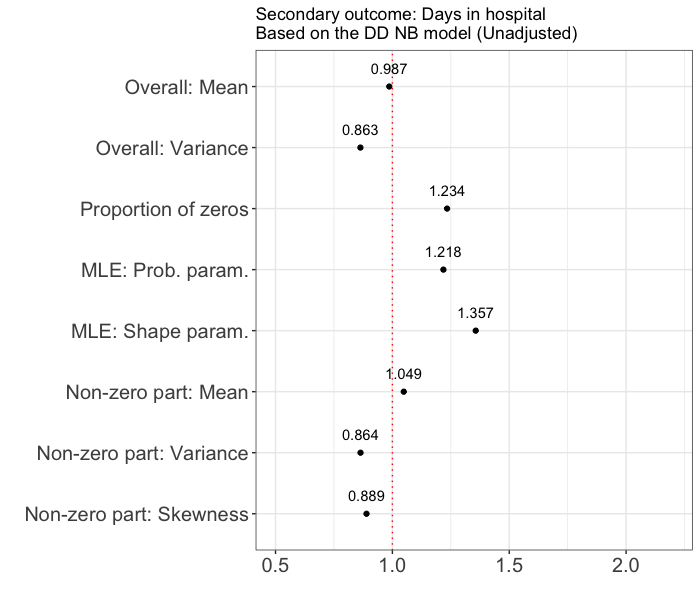


Supplementary Figure 14 From the DD NB model: Adjusted (left) and unadjusted (right) odds ratio for a preference for a ZINB model (over an NB model in terms of AIC) with the secondary outcome (days in hospital)


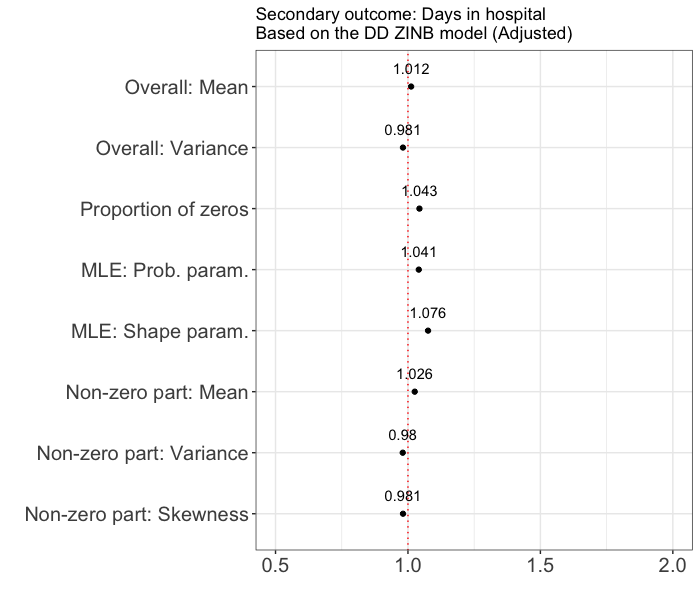

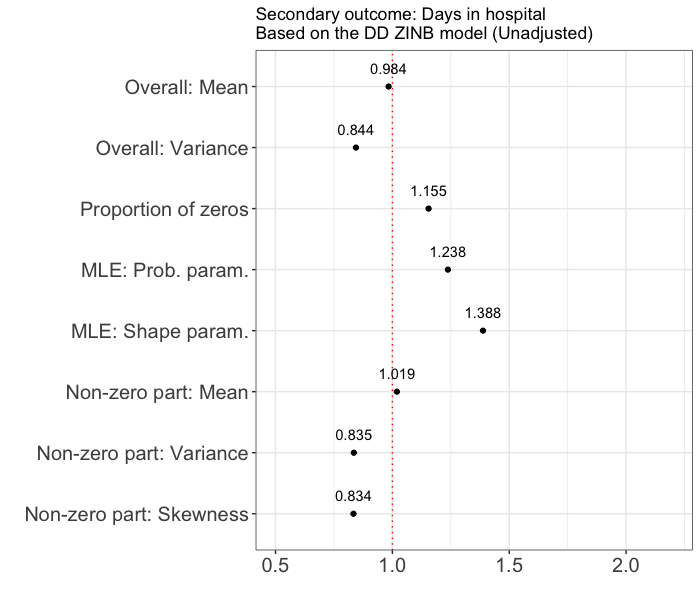


Supplementary Table 8 Unadjusted odds ratios with 95% confidence intervals (CIs) based on the unstandardized (raw) predictors regardless of the type of DD models. Asterisk indicates p-value < 0.05.

|  | **Serious illness episodes: Odds ratios (95% CIs)** | |
| --- | --- | --- |
| **Overall: Mean** | 1.38* | (1.09, 1.75) |
| **Overall: Variance** | 0.98 | (0.95, 1.01) |
| **Proportion of zeros** | 1.00 | (0.99, 1.01) |
| **MLE: Prob. param.** | 3.83* | (2.10, 6.96) |
| **MLE: Shape. param.** | 18.84* | (9.91, 35.82) |
| **Non-zero part: Mean** | 1.36* | (1.18, 1.57) |
| **Non-zero part: Variance** | 0.97* | (0.96, 0.99) |
| **Non-zero part: Skewness** | 0.80* | (0.76, 0.85) |

|  | **Care days outside the home: Odds ratios (95% CIs)** | |
| --- | --- | --- |
| **Overall: Mean** | 0.99 | (0.96, 1.01) |
| **Overall: Variance** | 1.00* | (1.00, 1.00) |
| **Proportion of zeros** | 1.64* | (1.55, 1.73) |
| **MLE: Prob. param.** | 8.65e+09* | (8.62e+05, 8.68e+13) |
| **MLE: Shape. param.** | 13.01* | (6.01, 28.18) |
| **Non-zero part: Mean** | 1.01 | (0.98, 1.03) |
| **Non-zero part: Variance** | 1.00* | (1.00, 1.00) |
| **Non-zero part: Skewness** | 0.86* | (0.77, 0.95) |

|  | **Days in hospital: Odds ratios (95% CIs)** | |
| --- | --- | --- |
| **Overall: Mean** | 1.00 | (0.97, 1.02) |
| **Overall: Variance** | 1.00* | (1.00, 1.00) |
| **Proportion of zeros** | 1.05* | (1.03, 1.07) |
| **MLE: Prob. param.** | 3.80e+16* | (7.65e+11, 1.87e+21) |
| **MLE: Shape. param.** | 8458.09* | (1412.28, 50655.01) |
| **Non-zero part: Mean** | 1.01 | (1.00, 1.02) |
| **Non-zero part: Variance** | 1.00* | (1.00, 1.00) |
| **Non-zero part: Skewness** | 0.90* | (0.86, 0.94) |

1. § Corresponding author: Kyung.Hyun.Lee@uth.tmc.edu [↑](#footnote-ref-1)
